# NLRP3 Inflammasome-Mediated Osteoarthritis: The Role of Epigenetics

**DOI:** 10.3390/biology14010071

**Published:** 2025-01-14

**Authors:** Yuzhou Liu, Ying Wang, Ping Yan, Ning Cui, Kejin Xu, Da Liu, Yuan Tian, Lingling Cao

**Affiliations:** 1 School of Pharmacy, Changchun University of Chinese Medicine, Changchun 130117, China; lyz616728@163.com (Y.L.); wang13053712756@163.com (Y.W.); xukj@ccucm.edu.cn (K.X.); 2 College of Traditional Chinese Medicine, Changchun University of Traditional Chinese Medicine, Changchun 130117, China; 22209559103@stu.ccucm.edu.cn (P.Y.); cuining@ccucm.edu.cn (N.C.); 3 Public Laboratory Centre, Changchun University of Chinese Medicine, Changchun 130117, China; liuda_1986@163.com; 4 Clinical School of Medicine, Changchun University of Traditional Chinese Medicine, Changchun 130117, China

**Keywords:** HDACs, NLRP3 inflammasome, HDAC inhibitors, osteoarthritis, chondrocyte pyroptosis

## Abstract

The prevalence of osteoarthritis (OA) notably surges with age and weight gain. The most common clinical therapeutic drugs are painkillers, yet they cannot impede the deteriorating course of OA. Thus, understanding OA’s pathogenesis and devising effective therapies is crucial. It is generally recognized that inflammation, *pyroptosis*, and OA progression are tightly linked. The activation of NLRP3 inflammasome can lead to the discharge of the pro-inflammatory cytokines Interleukin-1β and IL-18, intensifying subsequent inflammatory reactions and promoting OA development. Conversely, the imbalance caused by deacetylase-regulated NLRP3 inflammasome underlies the chronic mild inflammation related to degenerative diseases. Therefore, this article expounds on the mechanism of OA pathogenesis and the role of histone deacetylases (HDACs) in NLRP3 inflammasome-triggered OA, and illustrates the application of HDAC inhibitors in OA, striving to provide more insights into novel OA treatment approaches.

## Simple Summary

This is a narrative review. The occurrence of osteoarthritis (OA) correlates with diverse cell death modalities like *apoptosis* and *autophagy*, where inflammatory factors are significant. Notably, OA can trigger the NLRP3 inflammasome, subsequently inducing the release of the pro-inflammatory factors Interleukin-1β and IL-18, augmenting downstream inflammatory reactions. HDAC modulates bone-related genes and extracellular signaling pathways, being involved in both osteogenesis and OA. HDACi promotes osteoblast maturation and steers stem cell differentiation, and the application of HDACi in OA treatment is a crucial avenue for prospective research.

## 1. Introduction

Osteoarthritis (OA) is a long-term degenerative disorder. Pathologically, OA is characterized by articular cartilage deterioration, subchondral bone hardening, synovial inflammation, and bony protuberance formation, primarily affecting the knee, hip, hand, and adjacent tissues [1,2]. OA is significantly influenced by factors such as age, gender, overweight, heredity, and articular damage [3]. Of these factors, overweight is a prevalent contributor to OA. Evidence suggests that adipose tissue expansion and chronic low-grade inflammation in obese individuals result in joint pain and swelling [4]. Additionally, obesity can overload articular cartilage, disrupt joint alignment, and cause muscle weakness [5]. It has been suggested that women have a greater incidence of OA and experience more pronounced symptoms than men do [6]. The occurrence rate of OA rises with age and is often accompanied by hormonal irregularities. The prevalence of OA is on the rise among women aged 45 to 64 years [6]. OA influences in excess of 360 million people across the globe. Eighty percent of those affected experience restricted mobility and 25 percent potentially experience physical disability due to OA. Furthermore, the incidence of OA continues to rise annually [7]. Existing nonsurgical therapies for OA include physiotherapy focused on proper exercise and weight reduction and pharmacological interventions, like nonsteroidal anti-inflammatory medications, analgesics, other medications. However, these treatments only alleviate pain and do not halt disease progression [8]. Surgical treatment is often the only clinical option for advanced-stage OA [1,9,10]. However, owing to variations in individual joint anatomy, patients undergoing joint replacement surgery may encounter increased surgical challenges and postoperative complications. Thus, surgical treatment requires the comprehensive consideration of multiple factors.

OA pathogenesis can be broadly categorized into impaired chondrocyte production, enhanced catabolism, and imbalances in extracellular matrix synthesis and degradation [11]. Chondrocytes, the primary cells in cartilage, undergo epigenetic alterations during OA development [12]. Histone acetylation and deacetylation play crucial roles in various epigenetic molecular functions [13,14]. Nucleosomes comprise DNA coiled around histone octamers [15]. Histones are basic proteins that are essential components of nuclear chromatin in chondrocytes; they possess numerous lysine-binding sites and are readily modified by acetylation [16]. Histone deacetylases (HDACs) and histone acetyltransferases (HATs) regulate the deacetylation and acetylation of histones, respectively. By influencing the chromatin structure, HDACs and HATs regulate gene expression, thereby maintaining the stable and dynamic balance of normal chondrocyte physiological functions [17]. NLRP3 is related to the nucleotide-binding oligomerization domain (NOD)-like receptor protein family, which is predominantly found in immune and some nonimmune cells. It is a key component of inflammasomes and triggers an inflammatory response upon activation. HDACs are enzymes that mainly remove acetyl moieties from histones and can influence *NLRP3* gene transcription by altering the chromatin state. The initiation of the NLRP3 inflammasome leads to the release of inflammatory factors (e.g., Interleukin-1β and IL-18) that can affect intracellular signaling pathways. The aforesaid signaling pathways may subsequently modulate the transcription of HDAC-related genes [18,19,20]. SIRT2, a component of the HDAC family, induces the deacetylation of NLRP3 [21,22,23,24]. FSIRT2, an element of the HDAC family, induces NLRP3 deacetylation [21,22,23,24]. Furthermore, the potential influence of SIRT1 regarding the lipopolysaccharide (LPS)-triggered activation of the NLRP3 inflammasome in trophoblast cells suggests that SIRT1 inhibition enhances the LPS-induced inflammatory signaling process, interleukin-1β expulsion and NLRP3 presentation [24,25,26,27]. More importantly, SIRT1 alleviates LPS-induced oxidative stress [22,28,29]. A prior investigation uncovered a new mechanism through which SIRT1 exerts its anti-inflammatory effects, pinpointing SIRT1 regulation as a potential therapeutic strategy for preventing inflammation-related postoperative complications in osteoarthritis [30].

NLRP3 inflammasomes, part of the NLR family, are activated by danger signals from PAMPs and DAMPs. Innate pattern recognition receptors (PRRs) detect and activate NLRP3 proteins. Subsequently, ASCs and caspase-1 are recruited and activate the precursors of Interleukin-1β and IL-18, producing their fully developed forms. These cytokines are released extracellularly, triggering an inflammatory response [31,32]. The host must strictly regulate inflammasome activity to prevent excessive cytokine production and cell death. Regulation typically occurs at the transcriptional and posttranscriptional levels [33,34]. Acetylation is associated with increased transcriptional activation, whereas deacetylation is correlated with transcriptional inactivation [35]. Studies indicate that NLRP3, Interleukin-1β, and IL-18 are abundantly present in the peripheral blood of individuals with OA, potentially contributing to OA development [36]. In particular, the activation of the NLRP3 inflammasome has been noted in the synovial tissues of both OA patients and animal models. This activation subsequently sets off the Toll-like receptor (TLR) and nuclear factor-kappa B (NF-KB) signaling pathways, resulting in synovial inflammation and worsening the progression of OA [36]. NLRP3 or caspase-1 expression suppression significantly reduces arthropathy in rat OA models [37]. However, OA pathogenesis and epigenetic regulation remain poorly understood [38,39]. Understanding OA pathogenesis and the role of HDACs in NLRP3 inflammasome-induced OA is clinically important, and the further exploration of new treatment approaches is warranted. This article elaborates on these aspects from the aforementioned perspectives.

## 2. OA Pathogenesis

**Figure 1:**
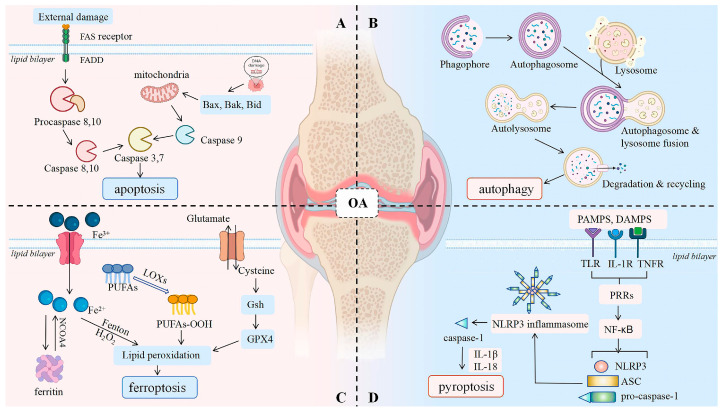
The pathogenesis of osteoarthritis. Sub-figure (**A**) illustrates the intrinsic apoptotic pathway. External damage activates the FAS receptor, which assembles a death-inducing signaling complex (DISC) with FADD and Procaspases 8 and 10. This subsequently activates Caspases 8 and 10, which in turn activate Caspases 3 and 7, initiating the apoptotic cascade. Bax, Bak, and Bid promote the release of cytochrome c from mitochondria, which then associates with Apaf-1 and Procaspase 9 to activate Caspase 9, thereby enhancing Caspase 3 activation and culminating in cell *apoptosis*. Sub-figure (**B**) depicts autophagy, wherein a phagophore engulfs cellular components, forming an autophagosome that subsequently fuses with a lysosome to generate an autolysosome for degradation and recycling. Sub-figure (**C**) illustrates *ferroptosis*, characterized by iron-dependent lipid peroxidation. Ferritin releases iron, which reacts with polyunsaturated fatty acids (PUFAs) to form PUFAs-OOH, resulting in lipid peroxidation. GPX4 and glutathione (GSH) mitigate this process, thereby preventing *ferroptosis*. Sub-figure (**D**) outlines *pyroptosis*, wherein the NLRP3 inflammasome activates Caspase-1, which processes pro-IL-1β and pro-IL-18 into their mature forms, triggering inflammation upon release.

Under normal conditions, chondrocyte secretion, proliferation, and differentiation remain relatively stable. However, the disruption of this balance leads to significant changes in the chondrocyte microenvironment. Various inflammatory factors directly or indirectly impact chondrocytes, resulting in abnormal cellular metabolism and secretion. A substantial decrease in chondrocyte numbers and accelerated matrix degradation disturb the balance between bone formation and osteoblastogenesis [40]. These changes ultimately result in cartilage tissue degeneration, epiphyseal formation, and eventual articular cartilage degeneration [41]. Notably, the pathogenesis of OA is intricately linked with chondrocyte *apoptosis* [42,43], *autophagy* [44,45], *ferroptosis* [46,47], and *pyroptosis* [47,48] (Figure 1).

### 2.1. Chondrocyte Apoptosis and OA

The excessive *apoptosis* of chondrocytes is a crucial factor in OA progression [49]. *Apoptosis* is initiated via two primary pathways: endogenous and exogenous. The intrinsic pathway is initiated via the oligomerization of the BCL-2 family proteins BAK and BAX. The extrinsic pathway is activated by the engagement of cell membrane receptors, such as tumor necrosis factor receptor 1 (TNFR1), death receptors, and Toll-like receptors (TLRs) [50]. Within the SD rat model, the administration of AICAR, an AMPK activator, was found to increase the expression of IL-10, IL-13, and BCL-2; reduce the levels of the NLRP3 inflammasome, TNF-α, IL-6, and Caspase-3; and increase the levels of HOIP, p-AMPK, IL-10, and IL-13. This treatment reduced NLRP3 inflammasome (including ASC, NLRP3, and Interleukin-1β), TNF-α, and IL-6 levels. These findings suggest that the LKB1/AMPK pathway significantly ameliorates the NLRP3 inflammasome reaction and chondrocyte damage [51]. In an OA mouse model, HUC-MSC-EVs have been shown to reduce osteoclast formation, increase COL2A1 expression, and aggregate proteoglycans in the knee joint. Additionally, HUC-MSC-EVs inhibited the overexpression of ADAMTS5 and MMP13 by reducing the secretion of proinflammatory factors [52]. TRAIL receptors (DR4 and DR5) are expressed by human articular chondrocytes, which regulate *apoptosis*, with elevated TRAIL and DR4 expression observed in OA rat cartilage [53]. In vivo investigations have shown that the LKB1/AMPK pathway markedly relieves the NLRP3 inflammatory reaction and chondrocyte damage. The activation of the AMPK pathway by the linear ubiquitination of LKB1 holds prospective therapeutic significance for treating OA [54,55].

### 2.2. Chondrocyte Autophagy and OA

Dysfunctional chondrocyte *autophagy* is a critical pathogenic mechanism in OA [56,57,58]. *Autophagy* is crucial for maintaining chondrocyte function, and OA progression is correlated with diminished autophagic activity [59,60]. *Autophagy* is a cellular recycling process in which partially damaged or aged cytoplasm and organelles are encapsulated in double-membrane vesicles and transported to lysosomes for degradation. These components are broken down into metabolites within lysosomes and recycled to meet the cell’s metabolic needs and facilitate organelle renewal [61,62]. In normal adult cartilage, moderate *autophagy* regulates chondrocyte maturation and modulates the terminal stage of the life cycle [63,64]. However, decreased autophagic activity leads to the buildup of impaired organelles and large molecules inside the cell. This accumulation impairs chondrocyte survival and differentiation, ultimately contributing to OA [65,66]. Studies have shown that inflammatory responses suppress chondrocyte proliferation and cell cycle progression, reducing autophagic rates [67]. The activation of the PI3K/AKT/mTOR signaling pathway, with the increased mRNA expression of Atg5 and Atg7 and elevated protein levels of LC3, Beclin1, and p62, has been found to suppress *autophagy* in OA rat articular chondrocytes [68]. Conversely, reducing COX-2, TNF-α, IL-6, and Interleukin-1β expression in OA has been discovered to activate the PI3K/AKT/mTOR signaling pathway. This activation inhibits chondrocyte autophagic activity, promoting OA progression [69]. Furthermore, in an OA mouse model, Nrf2 pathway suppression led to knee joint cartilage degradation and increased the serum levels of the inflammatory cytokines Interleukin-1β and IL-6. This reduction in autophagic activity accelerated OA progression [70]. Additionally, SIRT3 overexpression restored Interleukin-1β-induced *autophagy*, suppressed the activation of the PI3K/Akt/mTOR pathway and lessened the degree of OA-triggered joint damage in rats [71]. Conversely, FBXO21 suppressed *autophagy* in rat chondrocytes by increasing Interleukin-1β, TNF-α, and LPS levels via the JUNB-FBXO21-ERK axis. This reduced chondrocyte anabolism and increased *apoptosis* and decomposition metabolism [72]. Therefore, during OA pathogenesis, reduced chondrocyte autophagic activity accelerates cartilage matrix degradation, impedes proliferation, and weakens the ability to eliminate senescent organelles and inflammatory factors. This effect results in cellular inflammatory responses, chondrocyte damage, and *apoptosis*.

### 2.3. Chondrocyte Ferroptosis and OA

*Ferroptosis*, regarded as a novel cell death modality, exerts a pivotal function in OA progression [73,74]. This process occurs in chondrocytes, resulting in cartilage damage and contributing to the kick-off and evolution of OA [75,76]. The working principles of cellular iron toxicity primarily involve dysfunctions in intracellular iron, amino acid, and lipid metabolism [77]. In OA, HIF-2α activation leads to abnormal iron metabolism and the increased susceptibility of chondrocytes to *ferroptosis*, exacerbating OA [78]. NCOA4 interacts with ferritin, enhancing lipid peroxide accumulation and causing alterations in chondrocyte iron homeostasis [79,80,81,82]. Reduced AMPK/Nrf2/HO-1 signaling pathway activity promotes ferritin deposition in chondrocytes, increasing OA-related pain sensitivity [83]. Gong et al. demonstrated that cardamonin (CAD) effectively alleviated inflammation, cartilage degradation, and *ferroptosis* triggered by interleukin-1β (Interleukin-1β) by modulating *ferroptosis* and the P53 signaling pathway, thus spotlighting its prospective therapeutic function in osteoarthritis (OA) [84]. However, the initiation of the Nrf2/Gpx4 signaling pathway significantly inhibits the modifications in iron content in chondrocytes caused by the overexpression of Gpx4, HO-1, FTH1, and Nrf2, alleviating cartilage damage and restraining OA progression [85]. Therefore, chondrocyte death induced by iron overload and lipid peroxidation significantly impacts OA development. *Ferroptosis* might serve as a therapeutic objective for treating OA.

### 2.4. Chondrocyte Pyroptosis and OA

Cellular *pyroptosis*, also known as cellular inflammatory necrosis, represents a type of programmed cell death. It presents as incessant cell swelling until the cell membrane ruptures. This event prompts the discharge of cell constituents and initiates a powerful inflammatory reaction [86,87,88]. NLRP3 inflammasomes are strongly associated with chondrocyte *pyroptosis* in OA, influencing its onset and progression [89,90,91]. The triggering of the PI3K/Akt/NF-KB and NLRP3/caspase-1/GSDMD signaling routes gives rise to inflammation and the excessive expression of *pyroptosis*-associated proteins, including NLRP3, caspase-1, GSDMD, and NF-KB p65. This overexpression accelerates chondrocyte *pyroptosis*, exacerbating OA pain [92]. In a lipopolysaccharide-induced rat OA model, elevated NLRP3, Interleukin-1β, and IL-18 levels exacerbated OA by activating chondrocyte *pyroptosis* and collagen synthesis via the NLRP3/caspase-1 pathway [93]. The initiation of the TLR4/NF-KB signaling pathway remarkably elevates the levels of inflammatory factors like TLR4, NF-KB, and Interleukin-1β. Meanwhile, the NLRP3/caspase-1/GSDMD pathway triggers the excessive expression of NLRP3, caspase-1, and GSDMD, underlining the significance of inflammation in propelling the development of OA [94]. Furthermore, miR-155 exhibits a high level of expression in OA chondrocytes induced by LPS. When miR-155 is downregulated, it potentially restrains chondrocyte *apoptosis*. This occurs as it targets SMAD2 to hinder the NLRP3/Caspase-1 pathway, consequently leading to a reduction in the levels of caspase-1, Interleukin-1β, and IL-18 [95]. In the OA cell model, chondrocytes exhibit increased *apoptosis*, lesion-associated preprotein expression, and Interleukin-1β and IL-18 levels. The upregulation of miR-219a-5p deactivates NLRP3 signaling and suppresses OA progression by targeting FBXO3 [96]. Furthermore, irisin, which is a myokine secreted when people exercise, has the ability to revive the Interleukin-1β-induced type II collagen expression in OA chondrocytes. It achieves this by impeding the PI3K/Akt/NF-KB pathway, reducing the expression of MMP-13 and ADAMTS-5, as well as subduing the activity of NLRP3/caspase-1. In this way, it mitigates chondrocyte necrosis [97]. One study demonstrated that treatment with stromal cell-derived factor-1 (SDF-1) and collagenase, which suppress the AMPK pathway, significantly diminishes the expression level of NLRP3 and caspase-1, biomarkers related to chondrocyte changes in OA patients [98]. Another study indicated that in OA, the ROS/NLRP3/GSDMD pathway is activated in chondrocytes, triggering ROS release. This activation accelerates NLRP3 inflammasome activation and GSDMD cleavage, releasing inflammatory-promoting cytokines like interleukin-1β and IL-18 and matrix hydrolases, and causing pyroptotic chondrocyte death. Thus, the NLRP3/caspase-1/GSDMD pathway promotes OA development and aggravates pain [99].

Consequently, *apoptosis*, *autophagy*, *ferroptosis*, and *pyroptosis* in chondrocytes are highly correlated with OA. The initiation and the triggering of the NLRP3 inflammasome in the classical *pyroptosis* pathway is a crucial factor that cannot be neglected. The NLRP3 inflammasome-mediated OA pathway has garnered significant attention from researchers, and aiming at the NLRP3 inflammasome could potentially form an efficacious approach to diagnosing and treating OA (as presented in Table 1).

## 3. HDACs Regulate NLRP3 Inflammasome-Mediated OA

**Figure 2:**
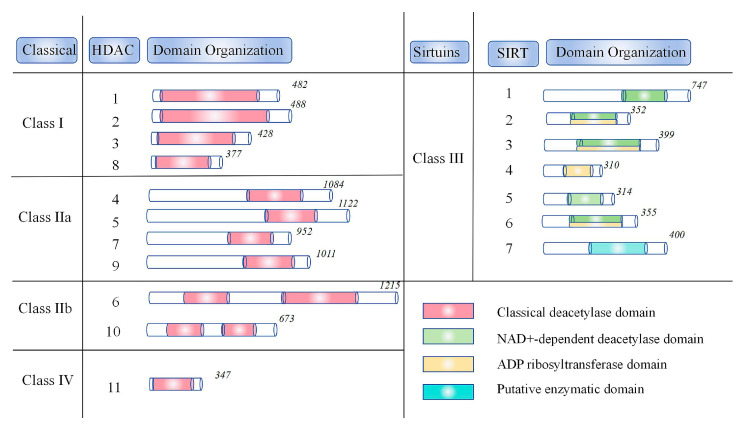
Specific classification of HDAC.

Histone acetylation and deacetylation are post-translational modifications in eukaryotic cells that are coregulated by HATs and HDACs [100]. Typically, histones in living organisms exist in an acetylated state. However, HDAC removes acetyl groups from histones. A low acetylation state results in reduced spacing between the histone and the DNA it encircles [101]. Currently, 18 HDAC isoenzymes have been identified. In light of their amino acid sequences and resemblance to the yeast genes Rpd3, Hdal, and Sir2, these genes are grouped into four categories: I, II, III, and IV (as shown in Figure 2) [102]. Class I HDACs are similar to the yeast Rpd3 protein and play crucial roles in cell growth, survival, DNA replication, and gene transcription. This class encompasses HDACs 1–3 and HDAC8. HDAC1 and HDAC2 are primarily nuclear, while HDAC3 and HDAC8 can move between the nucleus and cytoplasm. Class II HDACs are akin to the yeast Hdal protein and exhibit tissue-specific expression. They are further divided into subclasses IIa and IIb. Class IIa HDACs, like HDAC4, HDAC5, HDAC7, and HDAC9, can shuttle between the nucleus and cytoplasm. Class IIb HDACs, such as HDAC6 and HDAC10, are mainly cytoplasmic. Class III HDACs resemble the yeast Sir2 protein and require cofactor-catalyzed deacetylation with nicotinamide adenine dinucleotide, potentially involving inflammation. Class III HDACs consist of seven isoforms: SIRT1, SIRT6, and SIRT7 are nuclear; SIRT2 is cytoplasmic; and SIRT3, SIRT4, and SIRT5 are mitochondrial. HDAC11, classified as class IV, is predominantly nuclear [103,104,105,106,107].

NLRP3, serving as a vital sensor within the innate immune system, is capable of sensing exogenous pathogenic intrusions and endogenous cellular harm. In response, it initiates the assembly of the NLRP3 inflammasome, a supramolecular complex responsible for activating caspase-1. The principal constituents of the NLRP3 inflammasome are threefold: firstly, NLRP3 functions to detect danger cues and enlist downstream entities; secondly, caspase-1 prompts the maturation of cytokines like Interleukin-1β and IL-18 and modifies adermin D to expedite cytokine liberation and *pyroptosis*; lastly, ASC (*apoptosis*-associated speck-like protein containing a caspase recruitment domain) bridges NLRP3 and caspase-1, facilitating their interaction [33]. It has been demonstrated that Lamtor1 can interact with NLRP3 as well as histone deacetylase 6 (HDAC6). Notably, HDAC6 serves to strengthen the connection between Lamtor1 and NLRP3, thereby triggering the activation of the NLRP3 inflammasome [20]. In macrophage and dendritic cell models, in the context of macrophage and dendritic cell studies, the HDAC1 inhibitor, namely 4-(diethylamino)-N-[6-(hydroxyamino)-6-oxohexyl]-benzamide (DHOB), has been discovered to play a significant role. When subjected to TLR4 stimulation or in the presence of mycobacterium tuberculosis infection, DHOB was shown to drive the maturation of Interleukin-1β and boost its production. This was accomplished by upregulating NLRP3 expression, elevating the levels of cleaved caspase-1, and facilitating ASC oligomerization. Concerning a murine model of tuberculosis, DHOB led to an augmentation in both Interleukin-1β production and NLRP3 expression. In contrast, HDAC2 was involved in modulating Interleukin-1β production, and it achieved this by triggering the activation of the NLRP3 inflammasome [108].

Articular chondrocytes typically reside in a resting or hypometabolic state. Joint injury and repeated utilization have the potential to induce damage and trigger the onset of the OA disease process [109,110,111]. OA shows the characteristics of articular cartilage thinning, chondrocyte proliferation, abnormal chondrocyte hypertrophy, chondrocyte matrix enzyme degradation, enhanced matrix calcification, subchondral bone thickening, and joint inflammation [112,113]. OA pathogenesis is mediated by the nuclear factor kappa B (NF-KB), which is also known as the nuclear factor of kappa light polypeptide gene enhancer in B-cells, along with the mitogen-activated protein kinase (MAPK), and the Janus kinase/signal transduction and activator of transcription (JAK—STAT) complex signaling pathways, which are triggered by proinflammatory mediators, including cytokines such as Interleukin-1β and TNF-α [114]. HDACs contribute to the activation of these signaling pathways and influence the assembly of the NLRP3 inflammasome, thereby mediating OA progression (as shown in Table 2).

### 3.1. Class I HDACs

HDAC1 and HDAC2 may regulate cartilage-specific gene expression in primary human chondrocytes [115]. Studies have shown that, in OA chondrocytes, HDAC1 and HDAC2 protein levels increase, whereas collagen levels decrease [115]. Furthermore, the expression level of HDAC1, a regulator of chondrocyte metabolism, decreases during the chondrogenesis of human mesenchymal stem cells (HMSCs). Additionally, the overexpression of miR-520d-5p promotes HMSC chondrogenesis and modulates chondrocyte metabolism by directly targeting HDAC1 [116]. The in vitro suppression of HDAC1 activity in cultured chondrocytes also reduces type II collagen expression, potentially by inhibiting the transcription of the Wnt-5a gene [137]. Moreover, miR-503-5p is positively correlated with HDAC2 expression, and its inhibition suppresses OA chondrocyte *apoptosis* by downregulating HDAC2 expression via the NLRP3 inflammasome [119]. Additionally, HDAC2 and HDAC4 can interact with key regulators of the chondrocyte phenotype, RUNX2 and SOX9, thereby hindering the phenotypic development of OA chondrocytes [138]. Notably, in HMSCs, miR-92a-3p specifically targets the expression of HDAC2, modulating cartilage development and homeostasis and significantly enhancing cartilage matrix expression [139]. Many studies on the role of HDAC3 in articular cartilage have centered on its expression and regulation in normal chondrocytes and how HDAC3 governs chondrocyte development and growth. In HDAC3-deficient chondrocytes, diminished Erk1/2 phosphatase expression leads to the excessive phosphorylation of Erk1/2 and its downstream target RUNX2, resulting in increased MMP-13 expression and aggravated damage to chondrocytes [120]. HDAC3-deficient chondrocytes exhibit the elevated expression of cytokines and matrix degradation genes, such as IL-6 and Mmp3. Enhanced cytokine signaling increases JAK signaling autoactivation and modifies the STAT and NF-KB pathways, thus restraining chondrocyte maturation and the paracrine activation of osteoclasts and bone resorption. HDAC3 regulates the Phlpp1 and AKT pathways, thereby governing chondrocyte development and proliferation [16]. Additionally, miR-326 significantly reduces the levels of inflammatory cytokines, including NLRP3, Caspase-1, GSDMD, ASC, Interleukin-1β, and IL-18, by targeting the HDAC3 and STAT1/NF-KB p65 signaling pathways [121]. Another study indicated that miR-193b-3p inhibits cartilage formation and metabolism in HMSCs by targeting HDAC3, facilitating H3 acetylation, and decreasing MMP13 expression [140]. Furthermore, HDAC4 and HDAC8 may be crucial upstream mediators of the MAPK-mediated regulation of Interleukin-1β-induced chondrolysis metabolism and degradation. HDAC4 and HDAC8 exacerbate OA development by modulating Interleukin-1β through the MAPK pathway, promoting chondrocyte degradation, and suppressing NLRP3 inflammasome activation [141]. Moreover, the suppression of class I HDACs (HDAC1, HDAC2, and HDAC3) inhibits cytokine-induced metalloproteinase expression in chondrocytes and exosomes, thereby impeding cartilage resorption [142].

### 3.2. Class II HDACs

Class II HDACs exhibit minimal deacetylase activity toward acetylated histones, although they possess highly conserved catalytic domains [143]. HDAC4 plays a pivotal role in regulating the hypertrophic phenotypic changes in chondrocytes. It suppresses the expression of Runx-2 and inhibits the hypertrophic transformation of chondrocytes [144]. The upregulation of HDAC4 expression has been shown to inhibit the expression of Runx-2 and MMP-13 while promoting the synthesis of COL2A1 and ACAN, thereby alleviating articular cartilage damage [124]. Moreover, miR-483-5p accelerates the progression of osteoarthritis (OA) by targeting HDAC4, leading to chondrocyte hypertrophy, extracellular matrix degradation, and angiogenesis in the subchondral bone [124]. Another study indicated that miR-381 overexpression suppresses HDAC4 during chondrogenesis and increases MMP13 and RUNX2 expression, thereby preventing excessive chondrocyte atrophy and facilitating cartilage degeneration, leading to OA [125]. Ning and colleagues reported that histone deacetylase 4 (HDAC4) inhibits extracellular matrix degradation in chondrocytes induced by interleukin-1β (IL-1β), and this inhibitory effect is partially mediated through the WNT3A/β-catenin signaling pathway [126]. HDAC4 expression has been shown to regulate the onset and progression of age-related osteoarthritis (OA). Specifically, it modulates chondrocyte hypertrophy, suppresses the expression of COL2A1, a critical cartilage matrix gene, and upregulates MMP-13, a gene involved in cartilage matrix degradation [127]. Additionally, HDAC4 overexpression reduces Interleukin-1β, Cox2, and iNos expression, enhances glycan expression, and partially inhibits Interleukin-1-induced NLRP3 inflammasome activation, thereby affecting OA chondrocyte catabolism. Furthermore, HDAC4 levels are significantly lower in adult versus young donor cartilage, increasing the risk of OA development [127]. The suppression of miR-365 specifically targets HDAC4. The reduced expression of interleukin-1β (IL-1β) upregulates the expression of MMP13 and COL10A1 genes, thereby mitigating the inflammatory response in osteoarthritis (OA) [128]. The overexpression of miR-222 can exacerbate pain in OA patients; miR-222 downregulates HDAC4 and MMP-13 protein levels, accelerates *apoptosis*, and triggers an inflammatory response, resulting in medial meniscus cartilage instability [145]. High HDAC7 expression in OA accelerates cartilage degradation by suppressing Interleukin-1β expression. This suppression increases MMP-13 expression and increases the risk of OA development [146]. The increased expression of miR-193b-5p is a key factor contributing to the enhanced inflammatory response in osteoarthritis (OA) and is associated with an elevated risk of OA progression. Specifically, the overexpression of miR-193b-5p directly reduces HDAC7 and suppresses the expression of MMP3 and MMP13, thereby exacerbating the inflammatory response in chondrocytes during OA [147]. Among the WNT proteins, WNT10A activates atypical WNT/Ca2+ signaling in senescent OA MSCs. Subsequently, WNT10A induces the phosphorylation of HDAC5 downstream of histones and nuclear export via Ca2+/CaMKII, enhancing the OA regenerative microenvironment [148].

### 3.3. Class III HDACs

Class III HDACs, otherwise named sirtuins, are a unique class of HDACs that depend on nicotinic adenine dinucleotides and are not inhibited by Zn2+-binding HDAC inhibitors (HDACis) [129,149]. Type III HDACs play a critical role in regulating various aspects of cellular metabolism. This includes DNA repair, the modulation of the inflammatory response, and the regulation of *apoptosis* [150]. The sirtuin family, a subfamily of HDACs comprising seven members, has an impact on chondrocyte homeostasis and osteoarthritis (OA) pathogenesis. Existing research has focused primarily on SIRT1, SIRT3, and SIRT6 [151,152,153]. The increased expression of SIRT1 promotes the vitamin D receptor (VDR)-mediated proliferation of articular chondrocytes and the synthesis of extracellular matrix proteins. Additionally, it inhibits cellular senescence and the senescence-associated secretory phenotype (SASP), thereby mitigating age-related diseases [130]. Safflower yellow (SY) may prevent cartilage degeneration in OA by protecting chondrocytes and inhibiting inflammation via the modulation of the NF-KB/SIRT1/AMPK pathway and endoplasmic reticulum (ER) stress [131]. The activation of the SIRT1/mTOR signaling pathway enhances autophagic flux. Consequently, it protects chondrocytes from *apoptosis* and extracellular matrix degradation while inhibiting the progression of osteoarthritis (OA) [132]. Increased SIRT1 expression in chondrocytes reduces ATF4 expression and inhibits the PERK/eIF2α/CHOP pathway, thereby alleviating oxidative stress-induced osteoarthritis (OA) [133]. The regulation of the NF- NF-KB/SIRT1/AMPK pathway and endoplasmic reticulum (ER) stress results in the TNF-α-induced upregulation of interleukin-1β (IL-1β), PTGS2, and MMP-13 expression while downregulating COL2A1 and ACAN expression. This mechanism protects chondrocytes, mitigates inflammatory responses, and prevents degenerative cartilage pathology in osteoarthritis (OA) [131]. Mei et al. showed that 17β-estradiol (17β-E2) enhances mitochondrial *autophagy* through the SIRT1-mediated AMPK/mTOR signaling pathway, thereby protecting chondrocytes and providing novel mechanistic insights for osteoarthritis treatment [154]. Activation of the TGF-β1/Smad2 pathway and the inhibition of the NF-KB signaling pathway via SIRT1 suppresses the expression of matrix-degrading enzymes, including MMP-3, MMP-13, ADAMTS-4, as well as inflammatory mediators such as iNOS and COX-2, in chondrocytes. This suppression promotes the overexpression of the chondroprotective protein type II collagen, thereby enhancing chondrocyte viability and function, mitigating extracellular matrix degradation, and preventing progressive cartilage degeneration in osteoarthritic (OA) joints [134]. SIRT3 plays a pivotal role in regulating mitochondrial biogenesis and *autophagy*, and is a key regulator implicated in the pathogenesis of degenerative diseases. SIRT3 has been shown to decrease Interleukin-1β via the activation of the PI3K/Akt/mTOR pathway, resulting in mitochondrial malfunction and chondrocyte degeneration, thereby intensifying damage in OA [71]. Mitochondrial quality control mediated by the AMPK–SIRT3 positive feedback loop in chondrocytes sustains intrachondrocyte homeostasis, thereby governing the deterioration of OA pathogenesis [155]. The activation of the AMPK/SIRT3 signaling pathway mitigates mitochondrial damage, alleviates oxidative stress, and upregulates the expression of SOD2 and OGG1, thereby improving mitochondrial DNA integrity and functionality in osteoarthritic (OA) chondrocytes [135]. Through the modulation of the SIRT6/NF-KB and Nrf2/NF-KB signaling pathways to activate SIRT6 and downregulate the expression of ADAMTS-4, MMP-13, COX-2, and iNOS, articular chondrocyte degradation is prevented, and the inflammatory response in osteoarthritis (OA) is mitigated [136]. The activation of the SIRT6/NF-KB pathway in osteoarthritic (OA) chondrocytes inhibits NF-KB activity. This reduction in NF-KB activity significantly attenuates the inflammatory response induced by interleukin-1β, as well as the degradation of type II collagen and glycosaminoglycans, thereby inhibiting the progression of osteoarthritis (OA) [135].

In summary, although distinct HDACs function differently in cartilage, they are significant in OA development. HDACs can directly modulate the histone acetylation levels in chondrocytes, thereby influencing their cellular activity and regulating cartilage extracellular matrix expression. Moreover, the NLRP3 inflammasome represents a promising therapeutic target in the pathogenesis of osteoarthritis (OA). Histone deacetylases (HDACs) influence the activation of the NLRP3 inflammasome through various predominantly inflammatory signaling pathways, thereby modulating the expression of inflammatory factors in osteoarthritis (OA) and mitigating mitochondrial dysfunction and oxidative stress in chondrocytes, ultimately alleviating osteoarthritis (OA) symptoms.

## 4. HDAC Inhibitors

**Figure 3:**
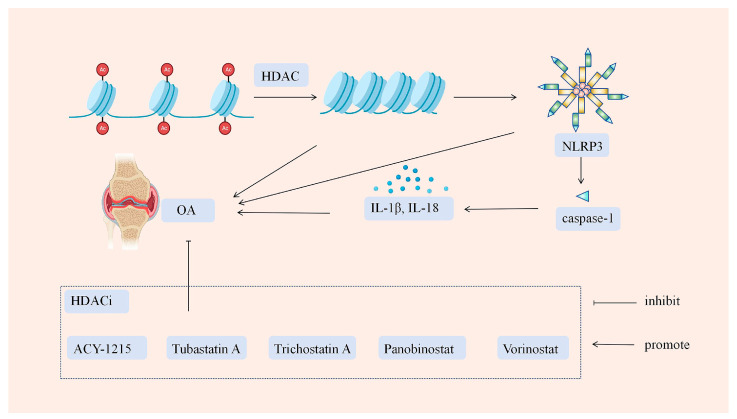
The role of HDACi in osteoarthritis.

Histone deacetylase inhibitors (HDACis) can be classified into four structural categories, specifically short-chain fatty acids, hydroxamic acids, aminobenzamides, and cyclic peptides [156]. Histone deacetylase inhibitors (HDACis) have been demonstrated to exert protective effects against osteoarthritis (OA) by preventing the specific interaction between histone deacetylases (HDACs) and histones, thereby modulating the histone acetylation levels [157]. HDACis are currently being evaluated as therapeutic agents for OA in various clinical trials, representing novel potential OA treatment targets for disease progression modulation, either through the direct inhibition of HDACs or the indirect obstruction of cellular sepsis resulting from NLRP3 inflammasome assembly (as shown in Figure 3).

ACY-1215 is recognized as a selective HDAC6 inhibitor, noted for its chondroprotective properties in osteoarthritis (OA), although its impact on subchondral bone has yet to be elucidated. In vitro studies have demonstrated elevated HDAC6 mRNA and protein expression in human OA osteoblasts. ACY-1215 inhibits the PI3K/AKT signaling pathway. Additionally, it downregulates the expression of vascular endothelial growth factor (VEGF) in osteoblasts and induces *apoptosis* in osteoarthritis (OA) osteoblasts in a concentration-dependent manner by activating the caspase pathway. When osteoblasts stimulated with ACY-1215 are cocultured with chondrocytes induced by interleukin-1β (IL-1β), the expression levels of MMP9 and MMP13 in chondrocytes are significantly reduced. Notably, the upregulation of HDAC6 expression in osteoblasts has been shown to promote osteoarthritis (OA) progression, suggesting that HDAC6 inhibitors hold promise as potential therapeutic agents for OA [158]. ACY-1215 has been documented to exhibit anti-inflammatory properties. It significantly suppresses the expression of inflammatory factors, including interleukin-1β (IL-1β) and interleukin-6 (IL-6), in human primary chondrocytes and C28/I2 cells. Furthermore, ACY-1215 may decelerate cartilage degradation by inhibiting the expression of matrix-degrading proteases, specifically MMP-1 and MMP-13, in chondrocytes, thereby exerting potent chondroprotective effects. These effects may be associated with the downregulation of the NF-KB and STAT3 signaling pathways mediated by ACY-1215 in osteoarthritis (OA) chondrocytes [159]. Tubastatin A, another HDAC6 inhibitor, effectively suppresses HDAC6 expression. It enhances cartilage integrity and prevents recurrent cartilage damage. Additionally, Tubastatin A efficiently suppresses HDAC6 expression, alleviates oxidative stress, decreases apoptotic protein levels, preserves chondrocyte viability, and inhibits extracellular matrix (ECM) degradation [160]. The inhibition of HDACs by trichostatin A (TSA) or butyrate (BA) results in a dose-dependent reduction in interleukin-1 (IL-1)-induced nitric oxide (NO) and prostaglandin E2 (PGE2) production. TSA and BA also inhibit NO and PGE2 production induced by interleukin-17 (IL-17) and tumor necrosis factor-alpha (TNF-α). This inhibitory effect is associated with the downregulation of inducible nitric oxide synthase (iNOS) and cyclooxygenase-2 (COX-2) protein and mRNA expression. Furthermore, TSA and BA suppress the IL-1-induced release of proteoglycans from cartilage-derived exosomes [161], Moreover, in vitro experiments utilized reverse transcription–quantitative polymerase chain reaction (RT–qPCR) to investigate changes in the mRNA expression levels of interleukin-1β (IL-1β) in TSA-treated chondrocytes. In vivo, TSA was administered via intra-articular injections to rats, followed by analyses of the mRNA and protein expression levels. Chondrocytes treated with IL-1β showed increased mRNA and protein expression levels of matrix metalloproteinases (MMPs) −1, −3, and −13, alongside reduced tissue inhibitor of metalloproteinases-1 (TIMP-1) mRNA and protein expression levels. Notably, these alterations were significantly mitigated by TSA treatment. Furthermore, increased MMP and decreased TIMP-1 expression levels were observed in an osteoarthritis (OA) rat model in vivo [162]. Panobinostat mitigated anterior cruciate ligament (ACL) rupture by suppressing the expression of histone deacetylases (HDACs) 4, 6, 7, and matrix metalloproteinase-13 (MMP-13). The inhibition of the FoxO transcription factor, which plays a critical role in maintaining the homeostasis of articular chondrocytes, may enhance proteoglycan 4 (PRG4) expression and reduce interleukin-1β (IL-1β)-induced inflammatory mediators and extracellular matrix-degrading enzymes. A reduction in these factors attenuates histological changes in the cartilage, synovium, and subchondral bone, while also alleviating pain-related behaviors [163,164]. Additionally, in the synovium of osteoarthritis (OA) rats, the knockdown of microtubule affinity-regulating kinase 4 (MARK4) inhibited the interleukin-1β (IL-1β)-stimulated interleukin-6 (IL-6) secretion and activation of the nuclear factor kappa-light-chain-enhancer of activated B cells (NF-KB) pathway in synovial mesenchymal stem cells (SMSCs). Furthermore, suberoylanilide hydroxamic acid (SAHA) reduced IL-1β-induced IL-6 secretion in SMSCs by inhibiting the MARK4/NF-KB pathway, thereby confirming a positive correlation among inflammatory factors in synovial samples [165]. Wang et al. demonstrated that histone deacetylase (HDAC) inhibitors, namely suberoylanilide hydroxamic acid (SAHA, vorinostat) and panobinostat (LBH589), significantly increased miR-146a expression. This effect was achieved by enhancing nuclear factor kappa-light-chain-enhancer of activated B cells (NF-KB) binding to the miR-146a promoter, while inversely regulating the interleukin-1β (IL-1β)-induced phosphorylation of IKK/IκB/p65 signaling and reducing the IL-6 secretion triggered by IL-1β [166] (as shown in Table 3).

## 5. miRNA Regulation of NLRP3 Inflammasome-Mediated Osteoarthritis

MicroRNAs (miRNAs), which are endogenous, small, noncoding RNAs approximately 22 nucleotides in length, regulate gene expression at the post-transcriptional level. They accomplish this through complementary base pairing with target mRNAs, thereby participating in various biological processes, including the regulation of inflammasomes [170]. The inflammasome, a cytosolic multiprotein complex, plays a crucial role in the inflammatory response. Upon cellular damage or infection, the inflammasome becomes activated, initiating an inflammatory response. In the context of osteoarthritis (OA), inflammasome activation leads to the release of inflammatory mediators such as interleukin-1 (IL-1) and tumor necrosis factor-alpha (TNF-α). These pro-inflammatory mediators promote chondrocyte *apoptosis* and extracellular matrix degradation, ultimately resulting in cartilage destruction and the progression of OA [171]. Numerous studies have demonstrated that mesenchymal stem-cell-derived extracellular vesicles (MSC-EVs) serve as potential drug carriers for the treatment of osteoarthritis (OA). This is achieved by targeting HDAC3 and STAT1/NF-KB p65, delivering miR-326 to chondrocytes and cartilage, and inhibiting cell death in cartilage [121]. Liang et al. [172] employed liposome membrane fusion to create hybrid EVs that target chondrocytes and encapsulate CRISPR/Cas9 plasmids. Their results indicated that these engineered EVs effectively targeted chondrocytes in cartilage injury, attenuated ECM protein degradation, and ultimately alleviated OA. Two studies have confirmed that miR-223 directly suppresses NLRP3 gene expression in myeloid cells, thereby reducing NLRP3 inflammasome activity and NLRP3-induced inflammatory responses, such as caspase-1 activation and interleukin-1β secretion, in bone marrow-derived macrophages, dendritic cells, and monocytes [173]. Liu et al. [174] have shown that human umbilical-cord-derived extracellular vesicles (HUC-EVs) enhance Interleukin-1β-induced chondrocyte survival and anabolism in vitro. In vivo experiments indicated that HUC-EVs mitigated MIA-induced inflammation and cartilage degeneration in OA rats. HUC-EVs were found to mediate the therapeutic effect of miR-223, which directly targets the 3′UTR of NLRP3 mRNA and inhibits NLRP3 inflammasome activation. HUC-MSC-derived EVs were demonstrated to slow OA progression by reducing the binding of miR-1208 to METTL3, decreasing the m6A level of NLRP3 mRNA, and inhibiting the secretion of proinflammatory factors. [52]. In vitro and in vivo experiments demonstrated that L-carnitine enhanced the anti-inflammatory effects of probenecid, attenuated MIA-induced OA, and reduced serum inflammatory factor levels by increasing miRNA-373 levels and inhibiting the P2X7/NLRP3/NF-KB pathway. The combination of these therapies had the most significant effect on improving OA symptoms [175].

## 6. Conclusions

OA pathogenesis involves *apoptosis*, *autophagy*, *ferroptosis*, and *pyroptosis* in chondrocytes, with inflammatory factors playing a significant role. More importantly, OA can initiate the NLRP3 inflammasome, leading to the release of the proinflammatory cytokines Interleukin-1β and IL-18, thereby enhancing downstream inflammatory responses. Conversely, the dysregulation of NLRP3 inflammasome acetylation underlies the chronic low-grade inflammation associated with degenerative diseases [51]. Therefore, understanding the role of HDACs in NLRP3 inflammasome-mediated OA is essential [108]. In recent years, HDACs have emerged as potential therapeutic targets for correcting the aberrant epigenetics associated with OA. Consequently, HDACs, as a novel research focus, offer new perspectives on the pathogenesis and therapeutic strategies for OA. However, many questions concerning their mechanisms of action remain to be explored. Histone deacetylation is a key aspect of epigenetic regulation, serving as a fundamental basis for cells to adopt diverse fates.

Furthermore, HDACs are the histone-modifying enzymes most extensively studied in orthopedics. They have been shown to specifically regulate bone-related genes and influence osteoblast differentiation. Moreover, HDACs regulate extracellular signaling pathways that influence bone formation and differentiation within the microenvironment. The functions of HDACs are multifaceted and complex, as they act on nonhistone proteins to deacetylate lysine residues. Many HDACs deacetylate a variety of proteins. For example, they deacetylate Runx2, a transcription factor related to bone formation. This modulation occurs either by removing the acetyl group from Runx2 or through direct physical interaction [176]. Most HDACs lack intrinsic chromatin-binding capacity; thus, they often function as transcriptional corepressors. Specific HDACs form complexes with bone-associated transcriptional regulators and modulate the expression of osteoblast-related genes, including Runx2, in the microenvironment [176]. Ma et al. demonstrated that the accumulation of HDAC6 in bone marrow stromal cells (BMSCs), accompanied by histone hypoacetylation at the Runx2 promoter in aged mice, led to a reduction in the osteogenic differentiation potential in vitro. Furthermore, the inhibition of HDAC6 was shown to activate Runx2 expression and enhance the osteogenic differentiation potential, thus mitigating bone loss in aged mice [177]. HDACs are not only directly associated with the osteogenic process of osteoblasts but also closely connected with OA. HDAC4 is directly involved in the osteogenic process and is a crucial factor in OA. HDAC4 suppresses the expression of Runx2 and Runx2-mediated MMP13 in chondrocytes from OA patients, as Runx2 influences chondrocyte hypertrophy [124,178]. Therefore, HDACs may regulate the pathogenesis of OA, although the in vivo function of HDACs in this disease remains uncertain. Notably, the inflammatory cytokines Interleukin-1β, IL-6, and IL-18 are highly expressed in OA. The NLRP3 inflammasome, which is upstream of these inflammatory cytokines, is a crucial target for cellular focus. Perichondral macrophages activate caspase-1 under the influence of DAMPs or PAMPs, leading to NLRP3 inflammasome activation. This results in the release of large amounts of interleukin-1β and interleukin-18, which increase the proinflammatory factor levels in chondrocytes. The recruitment of inflammatory factors stimulates chondrocytes to secrete catabolic enzymes, such as MMP13, leading to cartilage destruction [179]. Therefore, the NLRP3 inflammasome may be involved in OA development, and HDACs might contribute to the pathogenesis and symptoms of OA by modulating the NLRP3 inflammasome pathway.

HDACs can promote osteoblast maturation. For example, trichostatin A (TSA) is frequently utilized in bone tissue engineering [167]. HDACs induce human mesenchymal stem cell (HMSCs) differentiation into osteoblasts, chondrocytes, and other cell types. Furthermore, HMSCs cultured in vitro with TSA increase the release of alkaline phosphatase (ALP) and promote mineralization, enabling their differentiation into mouse cranial cells [162]. Moreover, valproic acid, another HDACi, inhibits the proliferation of human adipose-derived stem cells (HADSCs) and human bone marrow-derived stem cells (HBMSCs) in a dose-dependent manner. Valproic acid can be utilized for in vivo bone engineering following pretreatment. Conversely, in in vitro experiments, HMSCs were cultured into pellets with or without TSA, and after 21 days of chondrogenic differentiation, the pellet weights, glycosaminoglycan content at the DNA level, and cartilage markers were assessed in both groups; TSA inhibited cartilage formation and was not involved in the cartilage tissue engineering of HMSCs [180]. Most HDACi experiments have been conducted in vitro; in vivo experiments are limited because age is a significant causative factor for OA, making it difficult to ascertain the success of modeling in aged rats. Despite various aspects requiring attention, the use of HDACs in OA remains a crucial avenue for future research.

Finally, the pathogenesis of OA, a chronic disease, is highly complex and diverse. Throughout disease progression, various pathogenic mechanisms, including continuous inflammatory response activation, the metabolic imbalance of articular chondrocytes, abnormal mechanical load distribution, and immune regulation disruption, are intertwined and overlap. Thus, it is crucial to accurately assess which specific mechanisms constitute OA at different critical points in its development. Accurately identifying the main drivers of OA progression at different key stages allows the selection of the most appropriate treatment according to each patient’s individualized characteristics. This strategy based on precise diagnosis and targeted therapy is the core of current and future OA treatment, significantly enhancing treatment effectiveness, optimizing prognosis, and improving patients’ quality of life, offering new hope for OA patients.

## Figures and Tables

**Table 1:** The correlation between chondrocyte death patterns and osteoarthritis.

Cellular Demise Mode	Pathway	Target	Mechanism of Action	Reference
*apoptosis*	LKB1/AMPK	Interleukin—10 and Interleukin—13, Interleukin-1β, Tumor Necrosis Factor-alpha and Interleukin-6	In vivo experiments demonstrated that the LKB1/AMPK pathway significantly diminished the NLRP3-mediated inflammatory response and chondrocyte injury, suggesting it as a prospective aim for OA treatment through linear ubiquitination of LKB1 to activate the AMPK pathway.	[51]
	miR-1208/METTL3	COL2A1, METTL3, m6A, MMP13	In a murine model of osteoarthritis (OA), extracellular vesicles derived from human umbilical cord mesenchymal stem cells (HUC-MSCs-EVs) impede the release of pro-inflammatory cytokines and the breakdown of the cartilage extracellular matrix (ECM). This occurs as miR-1208 associates with METTL3, which in turn targets NLRP3 mRNA and diminishes its m6A methylation level.	[52]
	TRAIL	caspase 3, caspase 8, PARP, DR5, DcR1	Human articular chondrocytes display TRAIL receptors, specifically DR4 and DR5, which regulate *apoptosis*. Furthermore, an increased expression of TRAIL and DR4 was observed in cartilage from OA rats.	[53]
	miR-146a/b, miR-132, miR-155, NF-KB	IL-1, TUMOR NECROSIS FACTOR	In vivo experiments demonstrated that the LKB1/AMPK pathway significantly ameliorated the NLRP3-mediated inflammatory response and chondrocyte damage. The activation of the AMPK pathway through the linear ubiquitination process of LKB1 presents a prospective therapeutic approach for osteoarthritis (OA).	[54,55]
*autophagy*	PI3K/AKT/mTOR	Atg5, Atg7, Beclin1, LC3, p62; COX-2, Tumor Necrosis Factor—alpha, Interleukin-6, Interleukin-1β	In vitro experiments showed that activating the PI3K/AKT/mTOR signaling pathway resulted in reduced levels of Atg5 and Atg7 mRNAs, while the expression of LC3, Beclin1, and p62 proteins increased.	[68]
	PI3K/AKT/mTOR	Interleukin-1β, SIRT3	In vivo experiments demonstrated that the overexpression of SIRT3 restored Interleukin-1β-induced *autophagy* and subsequently re-inhibited the activation of the PI3K/Akt/mTOR signaling pathway.	[71]
	JUNB-FBXO21-ERK	Interleukin-1β, TUMOR NECROSIS FACTOR-α	In vitro experiments revealed that FBXO21 inhibited *autophagy* in rat chondrocytes by upregulating the levels of Interleukin-1β, TNF-α, and LPS through the JUNB-FBXO21-ERK axis.	[72]
*ferroptosis*	AMPK/Nrf2/HO-1	HO-1, Nrf2	The triggering of the AMPK/Nrf2/HO-1 signaling pathway facilitates ferritin deposition in a chondrocyte model.	[83]
	P53	Collagen II, p53, GPX4, MMP13, iNOS, COX2	In vitro studies demonstrated that the P53 signaling pathway reduced the expression of collagen II, p53, and GPX4, while upregulating MMP13, iNOS, and COX2 in chondrocytes.	[84]
	Nrf2/Gpx4	Gpx4, HO-1, FTH1, Nrf2	*Ferroptosis* in chondrocytes, induced by the overexpression of GPX4, HO-1, FTH1, and Nrf2, is inhibited by triggering the Nrf2/GPX4 signaling pathway in ex vivo experiments.	[85]
*pyroptosis*	NLRP3/caspase-1	NLRP3, Interleukin-18, Interleukin-1β	Increased concentrations of NLRP3, Interleukin-1β, and IL-18 proteins were noted as a result of the activation of the NLRP3/caspase-1 signaling pathway in a rat model of osteoarthritis (OA).	[93]
	TLR4/NF-KB, NLRP3/caspase-1/GSDMD	TLR4, NF-K Toll—like receptor 4, Nuclear Factor—kappa B, Interleukin-1β, NLRP3, caspase-1, GSDMD	In ex vivo experiments, the triggering of the TLR4/NF-KB signaling pathway led to a marked rise in TLR4, NF-KB, and Interleukin-1β inflammatory factors. Activation of the NLRP3/caspase-1/GSDMD signaling pathway induced the overexpression of NLRP3, caspase-1, and GSDMD pyroptotic proteins.	[94]
	NLRP3/Caspase-1	SMAD2, Cysteine protease caspase-1, interleukin-18, Interleukin-1β	In ex vivo experiments, the down-regulation of the miR-155 gene inhibited chondrocyte *apoptosis* by targeting SMAD2, which then restrained the NLRP3/Caspase-1 pathway and reduced the amounts of Caspase-1, Interleukin-1β, interleu-kin-18.	[95]
	FBXO3	Interleukin-1β, interleukin-18	In an osteoarthritis (OA) cell model, the upregulation of miR-219a-5p inactivates NLRP3 signaling and inhibits the progression of OA by targeting FBXO3.	[96]
	PI3K/Akt/NF-KB, NLRP3	Interleukin-1β, MMP-13, ADAMTS-5	In ex vivo assays, suppressing the PI3K/Akt/NF-κB signaling pathway managed to reinstate the expression of type II collagen, which had been downregulated by Interleukin-1β, in OA chondrocytes. Meanwhile, it lessened the expression levels of MMP—13 and ADAMTS-5, and curbed the activity of NLRP3/caspase-1 as well. Consequently, cartilage necrosis in OA chondrocytes was mitigated.	[97]

**Table 2:** The role of HDAC in osteoarthritis.

Classify	Target or Signaling Pathway	Mechanism of Action	Reference
Class I	HDAC1, HDAC2	HDAC1 and HDAC2 protein levels were elevated, which correlated with reduced collagen expression to varying degrees.	[115]
	HDAC1	Overexpression of miR-520d-5p enhanced the chondrogenic differentiation of human mesenchymal stem cells (hMSCs) and modulated chondrocyte metabolism by targeting histone deacetylase 1 (HDAC1).	[116]
	HDAC1, HDAC2	The transcription factor Snail was found to suppress type II collagen expression in chondrocytes via interacting with the carboxy-terminal functional domains of HDAC1 and HDAC2.	[117]
	HDAC1, Wnt-5a	HDAC1 might suppress the transcription of the Wnt-5a gene, consequently inhibiting type II collagen expression.	[118]
	NLRP3, HDAC2, MicroRNA-503-5p	Inhibition of miR-503-5p expression reduced osteoarthritis (OA) chondrocyte *apoptosis* by downregulating HDAC2 expression through NLRP3 inflammasomes.	[119]
	HDAC2, HDAC4, RUNX2, SOX9	HDAC2 as well as HDAC4 engage in interactions with RUNX2 and SOX9, both of which serve as crucial regulators for the chondrocyte phenotype. In doing so, they hinder the phenotypic progression of chondrocytes in osteoarthritis (OA).	[117]
	HDAC2	In human mesenchymal stem cells (hMSCs), miR-92a-3p directly aims at regulating HDAC2 expression for the modulation of cartilage development and homeostasis.	[117]
	HDAC3	The diminished expression of Erk1/2 phosphatase, coupled with the excessive phosphorylation of Erk1/2 and its downstream target RUNX2, led to a rise in the expression of MMP-13.	[120]
	HDAC3	Regulation of the Phlpp1 and AKT pathways in cartilage development.	[16]
	HDAC3, STAT1/NF-KB p65	Inhibition of inflammatory cytokine levels associated with *pyroptosis*.	[121]
	HDAC3	Promotes H3 acetylation, reduces MMP13 expression, and inhibits chondrogenesis and metabolism in human mesenchymal stem cells (hMSCs).	[122]
	HDAC4, HDAC8	The modulation of Interleukin-1β can facilitate chondrocyte degradation and suppress the activation of the NLRP3 inflammasome.	[123]
Class II	HDAC4	Inhibited the expression of Runx-2 and MMP-13 while augmenting the synthesis of COL2A1 and ACAN.	[124]
	HDAC4, miR-483-5p	miR-483-5p accelerates the development of osteoarthritis (OA) through its targeting of HDAC4. In doing so, it drives chondrocyte hypertrophy, leads to extracellular matrix degradation and spurs subchondral bone angiogenesis.	[124]
	HDAC4	Decreased the concentrations of Runx-2 and MMP-13, and type X collagen, thereby alleviating joint cartilage damage.	[125]
	HDAC4	Upregulating MMP-13 expression to regulate chondrocyte hypertrophy.	[126]
	HDAC4	Interleukin-1β blockade caused by NLRP3 inflammasome activation affects the catabolism of osteoarthritis chondrocytes.	[127]
	HDAC4	Overexpression of HDAC4 downregulated Interleukin-1β, COX-2, and iNOS expression, promoted glycosaminoglycan expression, and partially suppressed Interleukin-1β induced activation of NLRP3 inflammasomes, thereby modulating the catabolism of osteoarthritis chondrocytes.	[127]
	HDAC4	Downregulation of Interleukin-1β promotes the transcriptional activity of MMP-13 and type X collagen genes, thereby mitigating the inflammatory response in osteoarthritis.	[128]
	HDAC7	Excessive expression of miR-193b-5p directly downregulates HDAC7 and further suppresses MMP3 and MMP13 expression, thereby exacerbating the inflammatory response of chondrocytes in osteoarthritis.	[129]
Class III	SIRT1	The upregulated expression of SIRT1 promotes the proliferation of articular chondrocytes mediated by VDR (vitamin D receptor) and boosts the synthesis of extracellular matrix proteins.	[130]
	NF-KB/SIRT1/AMPK	Guards against cartilage degeneration in osteoarthritis by modulating the NF-KB/SIRT1/AMPK pathway and endoplasmic reticulum stress. In doing so, it safeguards chondrocytes and suppresses inflammation.	[131]
	SIRT1/mTOR	Enhancement of autophagic flux to protect chondrocytes from *apoptosis*.	[132]
	SIRT1, PERK/eIF2α/CHOP	Upregulation of SIRT1 expression inhibits ATF4 expression and suppresses the PERK-eIF2α-CHOP pathway, thus alleviating oxidative stress-induced osteoarthritis.	[133]
	NF-KB/SIRT1/AMPK	Regulation of the NF-KB/SIRT1/AMPK signaling pathway along with management of endoplasmic reticulum stress leads to the TNF-α-prompted upregulation of Interleukin-1β, PTGS2, and MMP-13, as well as the downregulation of COL2A1 and ACAN.	[131]
	SIRT1, TGF-β1/Smad2	Triggering the TGF-β1/Smad2 signaling pathway and restraining the NF—KB signaling pathway by means of SIRT1 brought about the repression of MMP-3, MMP-13, ADAMTS-4, iNOS, and COX-2 expression in chondrocytes.	[134]
	SIRT3, PI3K/Akt/mTOR	SIRT3 curtails the expression of Interleukin—1β by activating the PI3K/Akt/mTOR signaling pathway, and this subsequently leads to mitochondrial dysfunction and chondrocyte degeneration.	[71]
	AMPK-SIRT3	The activation of the AMPK/SIRT3 signaling pathway mitigates mitochondrial damage and enhances the expression of SOD2 and OGG1.	[135]
	SIRT6/NF-KB, Nrf2/NF-KB	The regulation of the SIRT6/NF-κB and Nrf2/NF-κB signaling pathways, as well as the activation of SIRT6, combined with the suppression of ADAMTS-4, MMP13, COX-2, and iNOS expression, significantly alleviated the inflammatory response associated with osteoarthritis.	[136]

**Table 3:** The role of HDACi in osteoarthritis.

HDACi	Pathway	Target	Mechanism of Action	Reference
ACY-1215	PI3K/AKT	HDAC6, Interleukin-1β, MMP-9, MMP-13,	In an in vitro study, mRNA and protein levels of HDAC6 were elevated in human osteoarthritis (OA) osteoblasts. Following ACY-1215 treatment, the PI3K/AKT signaling pathway was inhibited, and that of vascular endothelial growth factor (VEGF) was downregulated in osteoblasts.	[158]
	NF-KB, STAT3	HDAC6, Interleukin-1β, IL-6, VEGF, Matrix metalloproteinase-1, Matrix metalloproteinase-13	In interleukin-1β (IL-1β)-induced models of human primary chondrocytes and C28/I2 cells, the secretion of inflammatory factors, including IL-1β and interleukin-6 (IL-6), was suppressed, the production of matrix metalloproteinases (MMPs) −1 and −13 was reduced, and the nuclear factor kappa-light-chain-enhancer of activated B cells (NF-KB) and signal transducer and activator of transcription 3 (STAT3) pathways were downregulated.	[159]
Tubastatin A	NA	HDAC6	In studies conducted both in vitro and in vivo, TubA inhibited HDAC6 expression, attenuated oxidative stress, reduced apoptotic protein levels, and inhibited extracellular matrix (ECM) degradation.	[167]
Trichostatin A	NF-KB	IL-17, TNF-α, IL-1	In an interleukin-1 (IL-1)-activated chondrocyte model, trichostatin A (TSA) and butyric acid (BA) suppressed the expression of interleukin-17 (IL-17) and tumor necrosis factor-alpha (TNF-α). Furthermore, TSA and BA inhibited the IL-1-induced release of proteoglycans from cartilage explants. Additionally, IL-1 increased the DNA-binding activity of nuclear factor kappa-light-chain-enhancer of activated B cells (NF-KB).	[161]
	NA	MMP-1, MMP-3, MMP-13, Interleukin-1β, TIMP-1	Both in vitro and in vivo, following interleukin-1β (IL-1β) treatment, the mRNA and protein expression levels of matrix metalloproteinases (MMPs) −1, −3, and −13 increased, whereas the mRNA and protein expression levels of tissue inhibitor of metalloproteinases-1 (TIMP-1) decreased. Notably, these alterations were significantly mitigated by trichostatin A (TSA) treatment.	[162]
Panobinostat	NA	HDAC4, HDAC6, HDAC7, MMP-13	In both in vivo and in vitro experimental studies, panobinostat enhanced the expression of *autophagy*-related genes and proteoglycan 4 (PRG4). Additionally, it suppressed both basal and interleukin-1β (IL-1β)-induced expression of inflammatory mediators and enzymes responsible for extracellular matrix degradation. In a rat model of osteoarthritis (OA), panobinostat modulates the expression levels of HDAC4, HDAC6, HDAC7, Runt-related transcription factor 2 (RUNX2), and matrix metalloproteinase-13.	[163,164]
Vorinostat (SAHA)	MARK4/NF-KB	Interleukin-1β, IL-6	In both in vivo and in vitro experiments, SAHA reduced the Interleukin-1β-induced secretion of IL-6 in synovial mesenchymal stem cells (SMSCs) by inhibiting the MARK4/NF-KB signaling pathway.	[168]
	IKK/IKB/p65	miR-146a, IL-6, Interleukin-1β	The upregulation of miR-146a expression by vorinostat in an Interleukin-1β-treated OA-FLS model resulted in the negative regulation of Interleukin-1β-induced IKK/IKB/p65 phosphorylation signaling and IL-6 secretion.	[169]

## Data Availability

Data sharing is not applicable to this article.
